# Depression care trajectories and sustainable return to work among long-term sick-listed workers: a register-based study (The Norwegian GP-DEP Study)

**DOI:** 10.1186/s12913-025-12406-4

**Published:** 2025-02-19

**Authors:** Heidi Marie Meling, Valborg Baste, Sabine Ruths, Norman Anderssen, Inger Haukenes

**Affiliations:** 1 Research Unit for General Practice, NORCE - Norwegian Research Centre, Årstadveien 17, 5009 Bergen, Norway; 2 Department of Global Public Health and Primary Care, University of Bergen, Årstadveien 17, 5009 Bergen, Norway; 3 National Centre for Emergency Primary Health Care, NORCE - Norwegian Research Centre, Årstadveien 17, 5009 Bergen, Norway; 4 Department of Psychosocial Science, University of Bergen, Christies Gate 12, 5015 Bergen, Norway

**Keywords:** Depression, General practice, Sick leave, Sustainable return-to-work, Mental health, Drug therapy, Psychotherapy, Specialized mental healthcare, Health services research, Large database research

## Abstract

**Background:**

Depressive disorders can negatively impact work life sustainability for affected individuals. Little is known about depression care trajectories and their association with sustainable return to work (SRTW) after long-term sick leave. This study aimed to identify depression care trajectories during the first three months of sick leave among long-term sick-listed workers with depression and investigate their associations with SRTW.

**Methods:**

**Design:**

Nationwide cohort study using linked data from Norwegian health and population registries. Study population: All inhabitants of Norway aged 20–64 from 1 January 2009 to 1 April 2011, who were diagnosed with depression in general practice, and had reached three months consecutive sick leave (*n* = 13 624, 63.7% women). Exposure: Depression care trajectories during the first three months of initial sick leave, identified using group-based multi-trajectory modeling. Types of depression care included were general practitioner (GP) consults, GP longer consults and/or talking therapy, antidepressant medication (MED), and specialized mental healthcare. Outcome: SRTW, measured by accumulated all-cause sickness absence days during two-year follow-up after initial sick leave, with cutoffs at 0, ≤ 30, and ≤ 90 days. Analysis: Gender stratified generalized linear models, used to investigate the associations between depression care trajectories and SRTW, adjusting for sociodemographic factors and sick leave duration.

**Results:**

Four depression care trajectory groups were identified: *“GP 12 weeks”* (37.2%), *“GP 2 weeks”* (18.6%), *“GP & MED 12 weeks”* (40.0%), and *“Specialist, GP & MED 12 weeks”* (8.7%). The *“GP 12 weeks”* group (reference) had the highest proportion attaining SRTW for both genders. Men in the “*GP 2 weeks*” group had a 12–14% lower likelihood for SRTW compared to the reference. Women in the *“Specialist,GP & MED 12 weeks 12 weeks”* group had a 19- 23% lower likelihood for SRTW compared to the reference.

**Conclusion:**

The association between depression care trajectories and SRTW varies by gender. However, trajectories involving follow-up by the GP, including both standard and longer consults and/or talking therapy over 12 weeks, showed the highest likelihood of SRTW for both genders. Enhancing GP resources could improve SRTW outcomes by allowing more frequent and longer consultations or talking therapy.

**Supplementary Information:**

The online version contains supplementary material available at 10.1186/s12913-025-12406-4.

## Background

Depressive disorders affect approximately 5% of the global adult population, with prevalence rates higher among women (6%) than men (4%) [1]. These disorders pose a major challenge to the sustainability of work life for those affected, due to emotional, somatic, and functional impairments that depression can cause [2], along with an increased risk of sickness absence, long-term absence, and premature exit from the workforce [3–7]. High recurrence rates of depression further complicate these challenges [8]. Past episodes of depression and other common mental disorders, along with a history of absenteeism, have been identified as predictors of sickness absence [9]. Additionally, about 20–30% of workers diagnosed and sick-listed with depression and anxiety disorders relapse with recurrent sickness absence after they return to work (RTW) [10–12], underscoring the impact of these disorders on work life sustainability.

Depression-related sickness absence incurs substantial costs at multiple levels of work life. Affected workers may miss out on work opportunities, while workplaces may experience production losses. Societally, depression-related sickness absence leads to increased welfare costs and additional burdens on the healthcare system. In 2010, the economic impact of mood disorders in Europe was estimated at €113.4 billion, with 63.4% attributed to the indirect costs of temporary and permanent occupational disability [13]. Optimizing depression care and promoting a sustainable return to work (SRTW) for workers suffering from depression offers several benefits, including increased productivity and healthy life-years [14]. A crucial step towards a more sustainable work life is improving our understanding of the various depression care trajectories and their association with SRTW for individuals on long-term sick leave with depression.

In Norway, as in many other countries, general practitioners (GPs) in primary healthcare are usually the first point of contact for professional depression care, typically providing treatment consisting of psychological therapy (or ‘talking therapy’) and/or pharmacological intervention [15]. In severe cases, GPs can refer their patients to specialized mental healthcare [15, 16]. The ‘stepped care principle’ – where treatment intensity is increased (‘stepped up’) if a patient does not improve – is generally applied, as endorsed by depression care guidelines [17]. While sick leave certification is commonly a part of GPs’ depression care in Norway, work participation is recognized for its health-promoting potential, which can improve recovery prospects through mechanisms like increased self-efficacy and providing meaningful everyday activity. Consequently, GPs are encouraged to limit sick leave for patients with mild to moderate depression [18]. Numerous studies have investigated the effects of various depression treatment interventions aimed at reducing sick leave and promoting RTW [19], with early psychological treatment interventions, such as cognitive behavioral therapy, proven to shorten the duration of depressive episodes and sick leave [20, 21]. However, to our knowledge, no research has yet examined which depression care trajectories are associated with sustainable RTW.

We had two objectives in this study: firstly, to identify depression care trajectories during the initial three months of sick leave among long-term sick-listed workers with depression; secondly, to investigate associations between these trajectories and SRTW, adjusting for sociodemographic variables and duration of initial sick leave.

## Methods

### Setting

In Norway’s universal healthcare system, all residents have access to primary and specialized healthcare services, including prescription drugs like antidepressants, covered by the National Insurance Scheme. The Regular General Practitioner Scheme, integral to the primary healthcare system, mandates that GPs provide equitable and coordinated health care services, such as sickness absence certification, to their enlisted patients [22]. Approximately 98% of the Norwegian population has a designated GP [23], and Norwegian GPs annually see 70% of their list patients, facilitating continuity in follow-up and management of chronic or recurrent diseases like depression [24]. GPs issue about 85% of sickness certifications [25], and serve as gatekeepers to specialized healthcare services and health-related social welfare schemes, such as sick pay, work assessment allowance, or disability pension. Full or partial sick leave grants full income coverage for up to 365 days; employers cover the first 16 days, while the Norwegian Labour and Welfare Administration covers day 17 up to 365 [26].

### Study design

We conducted a nationwide registry-based cohort study using data from the Norwegian GP-DEP Study, which investigates pathways of depression care in general practice [27].

### Data sources

Data from national health and welfare registries for the period 2008–2013 were linked at the individual patient level using the encrypted unique personal identification number assigned to all residents of Norway. Data were stored and analyzed on a secure server at the University of Bergen.

We retrieved demographic data (i.e., birth year, gender, income, and occupation group) from *The National Population Register*. Information about individual’s educational attainment was obtained from *The National Education Database*. *The Norwegian Social Insurance Database (FD-Trygd)* provided data on start and end dates for social welfare benefits issued and covered by the Norwegian Labor and Welfare Administration. From this database, we obtained start and end dates for issued sick pay, work assessment allowance, disability pension, and retirement pension. *The Control and Reimbursement of Health Care Claims (KUHR)* database holds data regarding all fee-for-service claims from public primary care providers. From KUHR, we obtained information on each daytime consults with GP; date of contact, diagnoses recorded according to the International Classification of Primary Care, 2nd version (ICPC-2), and reimbursement codes for therapeutic measures recorded by GPs. Information on all prescription drugs dispensed to individual patients treated in ambulant care is stored by *The Norwegian Prescription Database (NorPD)*. NorPD provided the date for each dispensation of an antidepressant drug (Anatomical Therapeutic Chemical (ATC) code N06A) that was reimbursed for the treatment of depression. *The Norwegian Patient Registry (NPR)* provided information regarding patient contact with specialized healthcare, with diagnosis according to the International Classification of Disease, 10th version (ICD-10), codes F32, F33, F34, or F41.2.

### Study population

The study population consisted of a cohort of workers who, upon inclusion, had been on three consecutive months of certified sick leave (initial sick leave), regardless of the sick leave grade, with a depression diagnosis in general practice. The cohort was followed for two years after the end of initial sick leave, which could last for up to 365 days. To establish the cohort, we initially selected all individuals in Norway aged 20–64 in the period between 1 January 2009 to 1 April 2011. Among these, we selected those who had a depression diagnosis recorded in general practice (ICPC-2 code P76 in KUHR) and a certified sickness certification reimbursement code with a depression diagnosis in KUHR. Finally, we selected those who had reached three consecutive months of certified sick leave according to FD-trygd (*N* = 14,042). Individuals who received work assessment allowance, disability pension, or early retirement pension along with sick pay during the initial sick leave spell were excluded (*n* = 292). Additionally, we excluded individuals who died (*n* = 103, 0.75%) and/or emigrated (*n* = 81, 0.59%) during the initial sick leave or during the two-year follow-up after end of initial sick leave. The final study population included 13,624 individuals (63.7% women).

### Identification of depression care trajectories

Using group-based multi-trajectory modeling (GBTM) – a finite mixture model estimating groups of distinct trajectories from longitudinal data [28] – we identified latent subgroups of individuals following similar trajectories of depression care. The trajectories were based on registered treatment every two weeks during the first three months of initial sick leave, equating to six time periods. The four types of depression care included in the trajectories were: *regular GP consults* (number); *GP longer consults* (> 20 min, yes/no) and/or *GP talking therapy* (yes/no); *antidepressant medication* (dispensed, yes/no), and treatment in *specialized healthcare* (combining outpatient and inpatient treatment, yes/no). The term ‘talking therapy’ generally includes various psychological treatments provided by GPs, such as supporting conversations, counseling, and more structured psychotherapeutic approaches like cognitive-behavioral therapy [29, 30]. In this study, the basis for this term was reimbursement code 615 in the KUHR database, where it is defined as “talking therapy by a GP with a duration of at least 15 min with patients with mental disorders. The conversation must deviate from a normal conversation about medical issues and be of a therapeutic nature” [31]. The 615 reimbursement code does not, however, differentiate between different types of ‘talking therapy.’

The regular GP consult variable was used as Poisson-distributed, while the other three depression care variables were binomially distributed; all variables were handled with a quadratic function over time. Individuals were assigned to the trajectory group where their posterior probability of membership was highest [28]. We evaluated five different models with from two to six trajectories. The evaluation was based on the fit statistics Bayesian Information Criteria (BIC), distribution of participants (group size) and average posteriori probabilities of assignment to trajectory groups, and relevance of the trajectory groups (Additional file 1). The BIC-value did not reach a minimum but did not change much from four to five trajectories groups, both model with four and five trajectories had satisfactory distribution of participants (> 5%) and average posteriori probabilities (> 70). When inspecting the four and five trajectory models we found that model with four trajectories ‘grouped’ all patients with medication in one trajectory, whereas the model with five trajectories split these patients in two trajectories. We did not find that this ‘split’ added relevant information as these two trajectories were somewhat similar. Thus, we deemed the model with four trajectories to have the best fit.

### Exposure variables

The four depression care trajectories identified in the GBTM were used as exposure variables for the regression analyses (see results section for description of the four trajecories).

### Outcome variables

Three outcome variables, *SRTW 0*, *SRTW* ≤ *30*, and *SRTW* ≤ *90,* were constructed for the regression analyses based on the accumulated number of all-cause sickness absence days following RTW during a two-year (730 days) follow-up period after the end date of initial sick leave [32]. These binary variables were differentiated based on cut-offs for accumulated days of all-cause sick leave registered in the ‘Norwegian Social Insurance Database’ during follow-up:

- *SRTW 0* = 1 (yes) included those with no sick leave days registered;
- *SRTW* ≤ *30* = 1 (yes) included those with 0 to ≤ 30 sick leave days registered; and
- *SRTW* ≤ *90* = 1 (yes) included those with 0 to ≤ 90 sick leave days registered.

The value 0 (no) for any of the three SRTW outcomes included those who did not attain SRTW as listed above, and/or those receiving work assessment allowance, disability pension, or retirement pension during follow-up. Participants attaining SRTW 0 or SRTW ≤30 would also meet the criteria for SRTW ≤90. We constructed these three SRTW outcomes with different levels of sickness absence allowance, reasoning that a population at risk of recurrent sickness absence may require some allowance for some sickness absence after RTW for their work to be sustainable [33]. We opted for the 0, ≤30, and ≤90-day cut-offs after a consulting with colleagues who have expertise in both clinical general practice and general practice research, deeming these intervals sufficient to establish different levels of SRTW.

### Covariates

We recoded *age* into five categories ‘20–29’, ‘30–39’, ‘40–49’, ‘50–59’, and ‘60–64’ years. *Education* reflected the highest level of completed education. We recoded 10 groups from the Norwegian Standard Classification of Education into five groups, corresponding to the International Standard Classification of Education (ISCED 2011)[34]: ‘ ≤ 10th grade ‘ (ISCED 0–2, pre-primary to lower specialized education); ‘High school dropout’ (subcategory of ISCED 2, lower specialized education plus the first year of upper specialized education); ‘High school graduate’ (ISCED 3–4, upper specialized and post-specialized non-tertiary education); ‘College/University (low)’ (ISCED 5–6, first stage of tertiary education); ‘College/University (high)‘ (ISCED 7–8, second stage of tertiary education). *Income level* was recoded into three categories: ‘Low ‘ = €16 800, ‘Medium’ = €16 801–33 600, ‘Medium high/high’ = € ≥ 33 601. Norwegian kroner (NOK) were converted to Euros (€) based on the currency conversion rates as of 11 May 2023). The International Standard Classification of Occupations (ISCO-08) structure was the basis for *Occupational category,* which we categorized as **‘**Skilled labour and manual work’ (skilled agricultural, forestry, and fishery workers; craft and related trade workers; plant and machine operators, and assemblers; and elementary occupations), ‘Clerical, service, and sales work’ (clerical support workers, and service and sales workers) ‘Managerial, academic, and technical work’ (technicians and associate professionals, professionals, managers, and armed forces occupations). *Duration of initial sick leave* was based on days of consecutive sick leave and categorized into three categories ‘ ≤ 6 months’ (1–180 days), ‘6–9 months’ (181–270 days) and ‘9–12 months’ (271–365 days).

### Statistical analysis

We examined the distribution of demographic and socioeconomic characteristics, as well as duration of initial sick leave, using descriptive statistics (n (%), total, and for men and women, respectively). Distribution of different benefits among participants *not* attaining SRTW were also examined. Since an individual could transfer between benefits during the two-year follow-up the propotions presented exceed 100%.

After identifying the depression care trajectory groups in the GBTM, we ran gender-stratified Poisson regression analyses with robust variance estimates to manage convergence issues [35]. These analyses estimated the relative risk (RR) of SRTW outcomes across depression care trajectories with 95% confidence intervals (CI). In the regression analyses, missing data on the variable *Occupational category* (9.6% missing, 14.1% among men, 7.0% among women, Table 1) were addressed by multiple imputation, generating 100 imputed datasets and pooling the results from these datasets into one inference [36]. The trajectory group ‘GP 12 weeks consults’ (see Results section) was used as the reference group in the regression models due to a higher proportion of group members reaching SRTW during follow-up. Three hierarchical models are presented: Model 1 is the unadjusted model; Model 2 includes sequentially added adjustments for the sociodemographic variables – age, education, income, and occupational category (estimates showed very small changes for each added variable, so the sociodemographic adjustments are presented combined); and Model 3, where duration of initial sick leave is added to Model 2. All analyses were conducted using the software STATA/SE V.17.0 for Windows, and the GBTM was conducted using the traj plugin (version March 17, 2021) [37].

**Table 1:** Characteristics of long-term sick listed workers with depression in Norway 2009–2011

	**Total**	**Men**	**Women**
**Age**	Mean (SD)	Mean (SD)	Mean (SD)
	42.0 (11.2)	43.1 (11.2)	41.4 (11.2)
	n (%)	n (%)	n (%)
	13 624	4951 (36.3)	8673 (63.7)
**Education level**			
≤ 10th grade	3038 (22.3)	1314 (26.5)	1724 (19.9)
High school (dropout)	1420 (10.4)	521 (10.5)	899 (10.4)
High school (graduated)	4441 (32.6)	1794 (36.2)	2647 (30.5)
College/University (low)	3785 (27.8)	943 (19.0)	2842 (32.8)
College/University (high)	808 (5.9)	317 (6.4)	491 (5.7)
Missing	132 (1.0)	62 (1.3)	70 (0.8)
**Income level**^**a**^			
Low	1451 (10.7)	373 (7.5)	1078 (12.4)
Medium	7633 (56.0)	2231 (45.1)	5402 (62.3)
Medium high/high	4534 (33.3)	2342 (47.3)	2192 (25.3)
Missing	6 (0.0)	5 (0.1)	1 (0.0)
**Occupational category **^**b**^			
Skilled labor and manual work	1977 (14.5)	1423 (28.8)	554 (6.4)
Clerical, service, and sales work	4767 (35.0)	967 (19.5)	3800 (43.2)
Managerial, academic, and technical work	5575 (40.9)	1863 (37.6)	3712 (42.8)
Missing	1305 (9.6)	698 (14.1)	607 (7.0)
**Duration of initial sick leave **^**c**^			
1–180 days (6 months)	5381 (39.5)	1899 (38.4)	3482 (40.2)
181 – 270 days (> 6–9 months)	2470 (18.1)	860 (17.4)	1610 (18.6)
271 – 365 days (> 9–12 months)	5773 (42.4)	2192 (44.3)	3581 (41.3)

^a^Income level: *Low* = *16 800 EUR, Medium* = *16 801 – 33 600 EUR, Medium high/high* =  ≥ *33 601 EUR. Norwegian kroner (NOK) converted to Euro (EUR) according to currency conversion rates per 11. May 2023*

^b^Occupational category**:** Based on the International Standard Classification of Occupations (ISCO-08) structure, categorized as *Skilled labor and manual work* = skilled agricultural, forestry and fishery workers, craft and related trade workers, plant and machine operators, and assemblers, and elementary occupations, *Clerical, service, and sales work* = clerical support workers, and service and sales workers, *Managerial, academic, and technical work* = technicians and associate professionals, professionals, managers, and armed forces occupations

^c^Duration of initial sick leave: Consecutive days of sick leave spell presented in 3-month intervals. Individuals were included in the study population at the point of three months consecutive initial sick leave (90 days)

## Results

### Study population characteristics

The study population included 13,624 patients (63.7% women). The mean age was 43.1 years for men and 41.4 years for women (Table 1). Men were predominantly high school graduates (36.2%), while women predominantly had lower-level college/university education (32.8%). A large proportion of men (47.3%) had medium–high/high income, whereas a medium income was most prevalent among women (62.3%). In terms of occupation, 37.6% of men were engaged in managerial, academic, and technical work, while 43.2% of women were employed in clerical, service, and sales work. The initial sick leave lasted 9 to 12 months for 44.3% of men and 41.3% of women.

### Depression care trajectory groups and their treatment characteristics

**Fig. 1:**
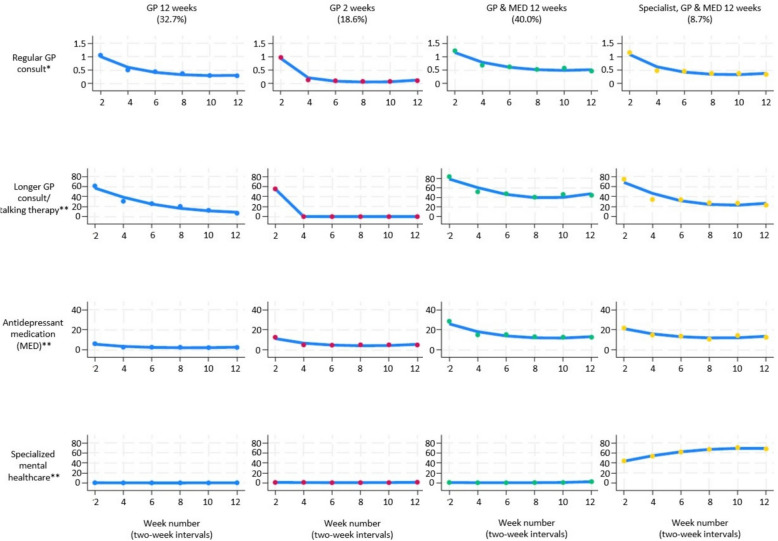
Depression care trajectories during the first three months of long-term sick leave. Trajectories of depression care among 13 624 long-term sick listed workers with depression in Norway 2009–2011. Depression care consisted of regular GP consults, longer GP consults and/or GP talking therapy, antidepressant medication (MED), or specialized mental healthcare measured every two weeks during the first three months of initial sick leave (12 weeks). The regular GP consult variable was used as Poisson-distributed, while the other three depression care variables were binomially distributed. * Number of regular GP consults per week, measured biweekly. ** Percentage (%) of group members receiving treatment, measured biweekly

Based on the group-based multi-trajectory model fit and evaluation of relevance (Additional file 1), we identified four distinguishable subgroups of individuals following similar trajectories of depression care during the first three months of initial sick leave due to depression, measured biweekly (Fig. 1). The trajectory group *‘GP 12 weeks’,* comprising 32.7% of the population, was characterized by members receiving GP consults througout the 12 weeks (Fig. 1). Members received one or more regular GP consultations during the first two-week period, and 60% of these were longer consultations and/or talking therapy. Subsequently, while GP consults continued, the proportion receiving regular consultations combined with longer consultation and/or talking therapy decreased to 4% in the last period. The *‘GP 2 weeks’* trajectory group (18.6% of the study population) had one consultation the first period, with about 56% being longer consultations and/or talking therapy, and about 12% of group members received medication. During the following periods, almost no further depression care was registered. Members of the *‘GP & MED 12* weeks’ trajectory group (40.0%) had one consultation in the first period, with approximately 85% being longer consultations and/or talking therapy; 40–50% had consultations with longer duration and/or talking therapy during the periods thereafter, and several group members also received medication; about 30% the first period, decreasing to 16%−13% in later periods. The final trajectory group, *‘Specialist, GP & MED 12* weeks’ (8.7%), was characterised by high and increasing rate of specialized mental healthcare, for 45–70% of members during the six periods. Most had a GP consultation in the first period – approximately 75% of these were longer consultations and/or talking therapy. Several members of this group also received drug treatment throughout the twelve weeks; 22% the first period, decreasing to around 10% during the last period. Members of the *‘Specialist, GP & MED 12* weeks’ group tended to be somewhat younger, had slightly higher education levels, and worked in managerial, acadamic and technical occupations (particularly women); they also had longer initial sick leave durations (Additional file 2).

### Depression care trajectories and sustainable return to work

The proportion of individuals who achieved SRTW within each trajectory group varied by gender and SRTW cutoff (Tables 2 and 3). Across all trajectory groups and both genders, the proportion attaining SRTW increased with increased allowance of sickness absence days during follow-up (0, ≤ 30, ≤ 90 days of sickness absence).

**Table 2:** Relative risk (RR) of sustainable return to work (SRTW) by depression care trajectory groups, men*

	**Model 1**		**Model 2**	**Model 3**
	SRTW**	Crude	+ age, education, income, occupation	+ duration of initial sick leave
	n (%)	RR (95% CI)	RR (95% CI)	RR (95% CI)
**SRTW 0**				
GP 12 weeks	564 (35.7)	1	1	1
GP 2 weeks	303 (29.8)	**0.83 (0.74–0.94)**	**0.84 (0.75–0.95)**	**0.86 (0.77–0.97)**
GP & MED 12 weeks	649 (32.9)	0.92 (0.84–1.01)	0.91 (0.83–1.00)	0.94 (0.86–1.03)
Specialist, GP & MED 12 weeks	123 (32.2)	0.90 (0.77–1.06)	0.87 (0.74–1.02)	0.93 (0.80–1.09)
**SRTW ≤ 30**				
GP 12 weeks	628 (39.9)	1	1	1
GP 2 weeks	342 (33.6)	**0.85 (0.76–0.94)**	**0.85 (0.77–0.95)**	**0.88 (0.79–0.97)**
GP & MED 12 weeks	736 (37.3)	0.94 (0.86–1.02)	0.93 (0.85–1.01)	0.96 (0.89–1.04**)**
Specialist, GP & MED 12 weeks	133 (34.8)	0.88 (0.75–1.02)	**0.84 (0.73–0.98)**	0.91 (0.78–1.05)
**SRTW ≤ 90**				
GP 12 weeks	773 (49.9)	1	1	1
GP 2 weeks	425 (41.8)	**0.85 (0.78–0.93)**	**0.86 (0.79–0.94)**	**0.88 (0.81–0.96)**
GP & MED 12 weeks	886 (44.9)	**0.92 (0.85–0.98)**	**0.90 (0.84–0.97)**	0.94 (0.88–1.00)
Specialist, GP & MED 12 weeks	162 (42.4)	**0.87 (0.76–0.98)**	**0.83 (0.73–0.95)**	0.91 (0.80–1.03)

Statistically significant estimates in bold

^*^ Sustainable return to work (SRTW) during two-year follow-up. SRTW outcomes have cutoffs allowing for 0, 0- ≤ 30, and 0- ≤ 90 days of accumulated sickness absence

^**^ Number and percentages within trajectory groups achieving SRTW outcomes (*N* = 4951)

**Table 3:** Relative risk (RR) of sustainable return to work (SRTW) by depression care trajectory groups, women*

	**Model 1**		**Model 2**	**Model 3**
	SRTW**	Crude	+ age, education, income, occupation	+ duration of initial sick leave
	n (%)	RR (95% CI)	RR (95% CI)	RR (95% CI)
**SRTW 0**				
GP 12 weeks	800 (30.4)	1	1	1
GP 2 weeks	525 (28.0)	0.92 (0.84–1.01)	0.93 (0.84–1.02)	0.93 (0.85–1.02)
GP & MED 12 weeks	904 (26.8)	**0.88 (0.81–0.96)**	**0.88 (0.82–0.96)**	**0.90 (0.84–0.98)**
Specialist, GP & MED 12 weeks	183 (23.1)	**0.76 (0.66–0.87)**	**0.74 (0.64–0.85)**	**0.77 (0.67–0.88)**
**SRTW ≤ 30**				
GP 12 weeks	950 (36.1)	1	1	1
GP 2 weeks	627 (33.5)	0.93 (0.86–1.01)	0.93 (0.86–1.01)	0.93 (0.86–1.01)
GP & MED 12 weeks	1073 (31.8)	**0.88 (0.82–0.95)**	**0.88 (0.82–0.95)**	**0.91 (0.85–0.97)**
Specialist, GP & MED 12 weeks	221 (27.8)	**0.77 (0.68–0.87)**	**0.75 (0.66–0.84)**	**0.78 (0.69–0.88)**
**SRTW ≤ 90**				
GP 12 weeks	1204 (45.7)	1	1	1
GP 2 weeks	810 (43.3)	0.95 (0.89–1-01)	0.95 (0.89–1.02)	0.95 (0.89–1.02)
GP & MED 12 weeks	1409 (41.8)	**0.91 (0.86–0.97)**	**0.92 (0.86–0.97)**	0.94 (0.89–1.00)
Specialist, GP & MED 12 weeks	291 (36.7)	**0.80 (0.72–0.89)**	**0.77 (0.70–0.86)**	**0.81 (0.73–0.89)**

Statistically significant estimates in bold

^*^ Sustainable return to work (SRTW) during two-year follow-up. SRTW outcomes have cutoffs allowing for 0, 0- ≤ 30, and 0- ≤ 90 days of accumulated sickness absence

^**^ Number and percentages within trajectory groups achieving SRTW outcomes (*N* = 8673)

Among men, the proportions attaining SRTW (0, ≤ 30, ≤ 90) were highest in the trajectory groups *‘GP 12 weeks*’,*‘GP & MED 12 weeks*’, and *‘Specialist, GP & MED 12 weeks*’ (Table 2). Members in* ‘GP 12 weeks*’ had the highest proportion and the largest increase in SRTW proportion when increased sickness absence days were allowed, ranging from 35.7% to 49.9%. The groups *‘GP & MED 12 weeks*’, and *‘Specialist, GP & MED 12 weeks’* did not differ significantly from the reference group *‘GP 12 weeks*’ with respect to the likelihood of achieving SRTW 0 and ≤ 30. Regarding SRTW ≤ 90, all trajectory groups had a lower likelihood of achieving SRTW compared to the* ‘GP 12 weeks*’, even after adjusting for age, education, income and occupation (Model 2). However, after final adjustment for the duration of initial sick leave, only ‘*GP 2 weeks*’ had a significantly lower likelihood of SRTW ≤ 90 (Model 3). Members of this trajectory group consistently had a lower chance of achieving SRTW compared to the reference group, with figures ranging from 12–14%, depending on the SRTW cutoff.

Among women, the proportions attaining SRTW (0, ≤ 30, ≤ 90) were highest in the two trajectory groups *‘GP 12 weeks*’ and *‘GP 2 weeks*’ (Table 3). All trajectory groups showed similar increases in the proportion attaining SRTW with increasing allowance for sickness absence days. For all SRTW outcomes, the two trajectory groups *‘GP & MED 12 weeks’* and *‘Specialist, GP & MED 12 weeks’* had a significantly lower likelihood of SRTW compared to *‘GP 12 weeks’* (reference) across all models of adjustment. The exception was *'GP & MED 12 weeks’*, which did not differ significantly from *‘GP 12 weeks*’ in attaining SRTW ≤ 90 after the final adjustment for the duration of initial sick leave (Model 3). The *‘Specialist, GP & MED 12 weeks’* trajectory had a 19–23% lower likelihood of SRTW compared to the reference, depending on the SRTW cutoff (Model 3).

The majority of the study population did not attain SRTW 0 (70.3%, 3,312 men, 6,261 women). Of these, a total of 63.3% were sick-listed, 46.3% were transferred to work assessment allowance, 5.9% received disablity pensions, and 1.9% received early retirement pensions (Table 4). A higher proportion of women were sick-listed than men (66.1% vs. 58.1%, respectively), while a somewhat larger proportion of men were transferred to other benefits.

**Table 4:** Proportion of benefits during two-year follow-up among participants who did not attain SRTW outcomes

	**Total**	**Men**	**Women**
	(*n* = 9573)	(*n* = 3312)	(*n* = 6261)
	n (%)	n (%)	n (%)
Benefits*			
Sick leave	6062 (63.3)	1924 (58.1)	4138 (66.1)
Work assessment allowance	4436 (46.3)	1648 (49.8)	2788 (44.5)
Disability pension	567 (5.9)	212 (6.4)	355 (5.7)
Retirement pension	177 (1.9)	119 (3.6)	58 (0.9)

^*^A person may transfer from one benefit to another during follow-up (proportions do not equal 100%)

## Discussion

### Main findings

In this register-based study of long-term sick-listed workers diagnosed with depression, four depression care trajectories were identified: *‘GP 12 weeks’*, *‘GP 2 weeks’, ‘GP & MED 12 weeks’*, and *‘Specialist, GP & MED 12 weeks*’. For both men and women, members of the *‘GP 12 weeks’* trajectory group (reference) had the highest proportion attaining SRTW. Among men, members of the *‘GP 2 weeks’* trajectory had a 12–14% lower chance of achieving SRTW compared to the reference. Among women, the *‘Specialist, GP & MED 12 weeks’* trajectory had a 19–23% lower likelihood of SRTW compared to the reference.

### Strengths and limitations

The primary strengths of this study lie in its large sample size and the linkage of comprehensive data from nationwide, high-quality health and administrative registers, which enabled the construction of precise measures of SRTW at the individual level during follow-up. Another strenght is the two-year follow-up period; most studies investigating SRTW limit their follow-up to a 1–3 month period post-RTW [38]. The use of data from the KUHR database, which records diagnoses of clinical depression, enhances the credibility of this study, as GPs typically make these diagnoses and provide treatment and sick leave certifications.

Regarding limitations, we lacked information on the severity of depression because ICPC-2 does not permit such grading. However, given that the study population had reached three months of consecutive sick leave with depression at inclusion, some homogeneity in severity can likely be inferred. We lacked information on graded sick leave in the Norwegian GP-DEP Study dataset used in this study. Graded sick leave could be indicative of a person’s level of functionality and might have served as a proxy for depression severity in the regression analysis. We encountered 9.6% missing occupational data, which was addressed using a multiple imputation procedure. While the initial sick leave duration exceeded six months for the majority of the study sample (60%), we focused our investigation of depression care trajectories within the first three months of sick leave. These early months are cruicial, as prompt intervention can improve treatment outcomes [39] and work-related outcomes such as regular work participation [40]. Moreover, NorPD contains data on dispensed medication, but we do not know whether patients used the drugs they collected, i.e. compliance. Since antidepressants are usually prescribed and collected for 3 months at a time in Norway, the 2-week prevalence of antidepressant treatment will likely be underestimated in this study. Mental and/or somatic comorbidity is common with depression [41, 42], and the dataset for the current study did not include data on additional diagnoses, making comorbidity a possible confounder. It is also important to note that this study population solely consisted of individuals long-term sick listed with depression, and thus, the findings are not generalizable to the general population. Norway’s generous social welfare system, providing full state-funded sick pay coverage for up to a year with only 16 days covered by employers, likely influences SRTW by providing limited incentives for workers and employers to reduce (and/or avoid) sickness absence [43]. Consequently, our findings may have limited generalizability but may still be of interest to other countries with universal healthcare and similar social welfare systems, such as the other Nordic countries. In neighboring countries such as Sweden, there was a marked shift in the frequency of sick leave diagnoses in the period between 2010 – 2020, where stress-related diagnoses (ICD-10 code F43: reaction to severe stress and adjustment disorder [44]) increased while depression diagnoses decreased [45]. This shift was, however, not observed in Norway during the same period; rather, the proportion of depression-related sick leave vs. what could be categorized as stress-related disorders remained relatively stable [46]. Because the association between depression care trajectories and SRTW and associations between stress-related care trajectories and SRTW, may look different, some caution in generalization of our results is to be encouraged, even within the Nordic countries.

### Findings in context

While studies have explored trajectories of depression symptoms [47, 48], this is, to the best of our knowledge, the first study to investigate depression care trajectories and sustainable return to work after long-term sick leave. We identified four trajectories reflecting varying levels of treatment type, intensity, and duration, aligning with the stepped care approach [17]. A majority of the study participants belonged to the depression care trajectories with a higher treatment intensity and duration (‘*GP 12 weeks’, ‘GP & MED 12 weeks’* and *‘Specialist, GP & MED 12 weeks’)*, indicative of significant symptom burden and lower level of functioning. This finding appears reasonable considering our study population was a cohort of workers long-term sick-listed for a minimum of three consecutive months, where 42.4% (44.3% of men and 41.3% of women) had an initial sick leave duration of nine to twelve months. Mental illnesses tend to cause the longest periods of sick leave in Norway; in 2022, the average duration was 73.9 days, an increase from 66 days in 2008 [49, 50], and in 2023, there was still a concerning trend of sick leave duration for mental illness extending even further [51]. Although we lacked information on severity of depression, long-term sick leave has been linked with severity of depression, and the duration of depression-related sick leave can serve as a measure for functionality [52]. Level of functionality is particularly relevant in the context of SRTW; it is viewed as a crucial aspect of remission by patients with depression [53] and plays a key role in GPs’ assessment of a patient’s work ability, which is a central factor in issuing sick leave certification for the issuance of welfare benefits such as sick pay. The processes of RTW and symptom reduction do not necessarily run parallel [54]. Helping patients maintain a level of functioning that allows for work participation could be a health-promoting and integral part of depression recovery that could make their return more sustainable. However, whether functionality can be maintained will also depend on the workplace’s opportunity and willingness to make necessary work accommodations [54].

Enhanced primary care depression management has previously been associated with positive work-related outcomes; a prior study found that enhanced care (i.e., treatment adherence support, psychoeducation, and more regular contact) for 24 months among employees with depression led to increased productivity and reduced absenteeism during a two-year follow-up period [55]. In the present study, the *‘GP 12 weeks’* trajectory group, which was characterized by members receiving regular GP consultations throughout the 12 weeks, had the highest proportion achieving SRTW (0, ≤ 30, and ≤ 90) among both men and women. However, this study indicates that more intensive GP care is not necessarily associated with a higher likelihood of SRTW, presumably due to a close relationship between the intensity of care and depression severity. Among women, this relationship seemed evident; the trajectory groups *‘GP 12 weeks’* and ‘*GP 2 weeks’* had the lowest intensity of care (e.g., no medication or specialized mental healthcare), but the highest proportion and likelihood of attaining SRTW.

Conversely, among men, members in the *‘GP 2 weeks’* trajectory group (i.e., the group with the lowest intensity of care) had the lowest proportion of SRTW attainment and the lowest likelihood of SRTW outcomes, compared to the *‘GP 12 weeks’* group (reference). Gender can play a critical role in health matters. Men have lower mental health service utilization [56], less positive attitudes towards mental health [57], and lower mental health literacy [58], defined as ‘the knowledge and abilities necessary to benefit mental health’ [59]. If men in the *‘GP 2 weeks’* group shared some of these gendered characteristics, it could have made them less likely to possess the attitudes, knowledge and abilities necessary to accept or adhere to depression treatment. However, this interpretation requires further empirical investigation, as the aforementioned characteristics were not a part of our data in the current study.

Furthermore, it is also possible that members of both genders in the *‘GP 2 weeks’* trajectory group were in contact with their GPs and received treatment for other conditions during the three-month observation period. This seems likely for several members, due to the high comorbidity rates. As part of the Norwegian GP-DEP Study, we recently conducted a nationwide registry-based cohort study among all Norwegian residents aged over 20 with a new depression diagnosis recorded in general practice (*n* = 307,237). The study revealed that 41.3% had comorbidity (27.0% somatic, 9.3% mental, and 5.0% both) [60]. It is also possible that some members of the *‘GP 2 weeks’* trajectory groups pursued treatment in low-threshold community programs or the private healthcare sector after their initial consultation, which would not be captured in the register data.

Interestingly, among women, the *‘GP 2 weeks’* group did not significantly differ from the *‘GP 12 weeks’* group (reference) in terms of the likelihood of attaining SRTW. Membership in this group among women could indicate less severe depression, thereby increasing the likelihood of attaining SRTW. It is also possible that women’s more active health-seeking behavior [61] guides them towards alternative treatment options such as community programs or private healthcare services. The *‘Specialist, GP & MED 12 weeks’* group consistently exhibited a lower proportion of SRTW attainment among women, had a higher proportion of longer initial sick leave durations, and a lower likelihood of achieving any SRTW outcome, compared to the reference group. Assuming the application of a stepped care approach, members in this group likely had a higher level of depression severity, which has been shown to be negatively linked to RTW outcomes [62].

## Conclusion

The associations between depression care trajectories and SRTW exhibit distinct patterns for men and women. The trajectory characterized by GP follow-up involving frequent and longer consults and/or talking therapy, appeared to be the most advantageous for both genders in terms of likelihood of SRTW. Thus, enhancing GP resources to allow for more frequent and longer consultations and/or talking therapy could improve SRTW outcomes. However, these findings should be read with caution, as depression severity is a likely confounder we could not control for. Extended periods of depression care observation, and mixed methods designs using both quantitative methods (e.g., medical records analysis) and qualitative methods (e.g., patient interviews) could provide a more comprehensive understanding of how depression care trajectories relate to sustainable return to work after long-term sick leave with depression in future studies.

## Supplementary Information

Supplementary Material 1: Additional file 1: Model fit for group-based multi-trajectory analysis of depression care; all order 2. Additional file 1: Table reporting measures of goodness of fit for group-based trajectory models. Additional file 2: Distribution of participant characteristics across depression care trajectory groups. Additional file 2: Table shows how participant characteristics are distributed across the four depression care trajectory groups.

## Data Availability

The data used in this study are provided by Statistics Norway, the Norwegian Directorate of Health, and the Norwegian Institute of Public Health, with restrictions only to be used under licence for researchers in the current study, thus, they are not publicly available. However, the registry data used in this study will be available from the authors upon reasonable request and with included permission from the Regional Ethical Committee for Medical and Health Research Ethics, Region West, Norwegian Data Protection Authority, Statistics Norway, the Norwegian Directorate of Health and the Norwegian Institute of Public Health.

## Abbreviations

AI: Artificial intelligence
ATC: Anatomical Therapeutic Chemical
BIC: Bayesian Information Criteria
CI: Confidence interval
FD-Trygd: Norwegian Social Insurance Database
GBTM: Group-based multi-trajectory modeling
GP: General practitioner
ICD: International Classification of Disease
ICPC: International Classification of Primary Care
ISCED: International Standard Classification of Education
ISCO: International Standard Classification of Occupations
KUHR: Control and Reimbursement of Primary Health Care Claims Database
MED: Medication
NOK: Norwegian kroner
NorPD: Norwegian Prescription Database
NPR: Norwegian Patient Registry
RR: Relative risk
RTW: Return-to-work
SRTW: Sustainable return-to-work
